# Congenital Oligodendroglioma: Clinicopathologic and Molecular Assessment with Review of the Literature

**DOI:** 10.1155/2015/370234

**Published:** 2015-02-10

**Authors:** Hope Richard, Kimberly Stogner-Underwood, Christine Fuller

**Affiliations:** ^1^Department of Pathology, Virginia Commonwealth University Health System, 1101 E. Marshall Street, P.O. Box 980662, Richmond, VA 23298, USA; ^2^Department of Pathology, Wake Forest Baptist Medical Center, Medical Center Boulevard, Winston-Salem, NC 27157, USA

## Abstract

Oligodendroglioma is an infiltrating glial neoplasm frequently seen in adults. Pediatric oligodendrogliomas are rare, with very few cases presenting in infancy and only rare congenital examples. In contrast to adult oligodendrogliomas, pediatric cases typically lack 1p/19q codeletion. Herein we report a case of WHO grade II oligodendroglioma diagnosed in a 7-month-old male infant. The patient initially presented at 3 months of age with symptoms suspicious for seizure. Initial workup including electroencephalography (EEG), electrocardiogram (EKG), and computed tomography (CT) of the head was negative. His symptoms persisted, and subsequent magnetic resonance imaging (MRI) performed at age of 7 months revealed a 2 cm contrast-enhancing left temporal lobe mass. The mass was excised and the microscopic appearance was that of a classic low grade oligodendroglioma composed of cells with uniformly round nuclei, perinuclear halos, delicate branching capillaries, and an absence of high grade features. Mutant specific (R132H) isocitrate dehydrogenase-1 (IDH1) immunohistochemistry was negative, and the tumor lacked detectable 1p or 19q deletions by fluorescent in situ hybridization (FISH). The onset of neurological symptoms in early infancy followed by the positive MRI findings suggests that this case represents a rare example of congenital oligodendroglioma.

## 1. Introduction

As a group, gliomas are the most common primary central nervous system (CNS) neoplasms in both adult and pediatric populations, whereas infiltrative gliomas, in particular glioblastoma, make up the bulk of adult glial tumors, pilocytic astrocytoma, and ependymoma predominate in children. Oligodendrogliomas may be encountered at any age; however, they are generally infrequent in the pediatric population. Congenital examples, those presenting at or shortly after birth, are even rarer. Here we describe a case we believe to represent a congenital oligodendroglioma, the patient initially presenting with seizure-like activity at three months of age.

## 2. Case Report

**Figure 1:**
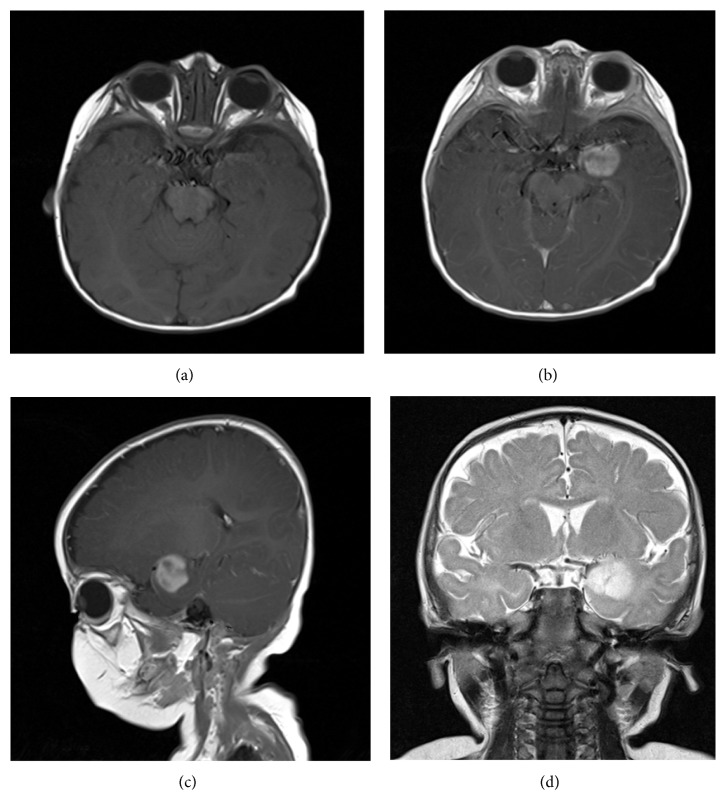
(a) Axial T1 precontrast, (b) axial T1 postcontrast, (c) sagittal T1 postcontrast, and (d) coronal T2 magnetic resonance imaging show a heterogeneously enhancing mass in the left temporal lobe.

A 3-month-old male infant was brought to the Emergency Department (ED) for evaluation of multiple episodes of presumed seizure activity. The episodes, which lasted 20–25 seconds, were described as staring spells accompanied by apnea and perioral cyanosis. A period of deep sleep followed, and upon waking the infant returned to baseline activity. No seizure activity was identified on electroencephalogram (EEG), and computed tomography (CT) of the head was negative for the presence of an intracranial abnormality. He was diagnosed with reflux and started on an antacid and specific feeding regimen. Seizure-like activity continued and follow-up magnetic resonance imaging (MRI) performed at 7 months of age revealed a 2 cm left temporal lobe lesion which was hyperintense on both T2-weighted (Figure 1(d)) and FLAIR images and isointense on T1 sequences (Figure 1(a)). The lesion enhanced heterogeneously with contrast (Figures 1(b) and 1(c)); however, no definite cysts or calcifications were detected. Gross total surgical resection of the mass was accomplished.

**Figure 2:**
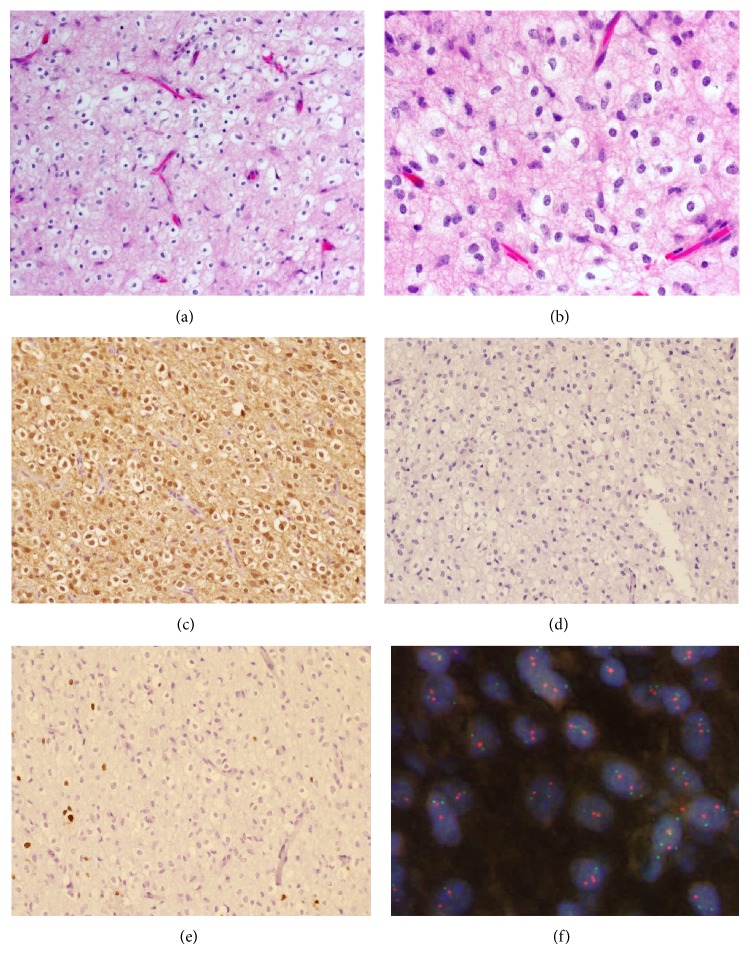
H&E stained sections at 20x (a) and 40x (b). Cytoplasmic and nuclear staining with S100 (c). Negative staining for mutant specific (R132H) IDH-1 (d). Low proliferation index (<1%) by Ki67 immunohistochemistry (e). Intact 1p and 19q by FISH (f).

Histologically, tumor consisted of a monomorphous population of cells with round, regular nuclei surrounded by clear perinuclear halos. Delicate, branching capillaries were interspersed throughout the tumor and calcifications were present focally. High grade features such as necrosis, microvascular proliferation, frequent mitoses, and nuclear pleomorphism were absent. The morphologic appearance was that of a classic low grade oligodendroglioma (Figures 2(a) and 2(b)). By immunohistochemistry, the tumor cells showed nuclear labeling for S100, with diffuse background staining for both S100 and glial fibrillary acidic protein (GFAP). Synaptophysin and mutant-specific isocitrate dehydrogenase-1 (R132H) immunohistochemistry were negative (Figures 2(c) and 2(d)). The Ki-67 labeling index was low (<1%) (Figure 2(e)). Fluorescent in situ hybridization (FISH) testing confirmed the absence of deletion of either chromosome 1p or 19q (Figure 2(f)).

Follow-up revealed that at five years of age the patient is clinically stable. A small focus of enhancement was identified on MRI at his one-year follow-up; however, this lesion has remained stable to date.

## 3. Discussion

As a group, glial neoplasms are by far the most common CNS tumors arising in the pediatric population, with astrocytomas and ependymomas representing the majority of these tumors. Oligodendrogliomas account for less than 3% of primary CNS tumors in children [1]. Congenital brain tumors are quite rare, the most common being teratoma, astrocytoma, medulloblastoma, choroid plexus papilloma, and ependymoma [2, 3]. Congenital oligodendroglial tumors are even rarer. Reported cases of congenital oligodendroglioma range from cases identified in utero [3] or within the first few days of life [2] to cases presumed to be congenital, with a tumor diagnosed in the first few months of life [4, 5]. Presenting signs and symptoms of these patients have included decreased fetal movement [3], seizure, headache, visual field defects, weakness, cranial nerve palsy [6], irritability [2], and apnea [4]. The report of one case noted an initial presentation of jaundice [2], thought to be secondary to hemorrhage within the tumor. The early onset of neurologic symptoms in the current case is most consistent with a congenital oligodendroglioma.

A number of alternate diagnoses should be considered when an oligodendroglial-like lesion is encountered in a child. These include neurocytoma, other gliomas such as astrocytoma and clear cell ependymoma, and dysembryoplastic neuroepithelial tumor (DNET). In the current case, S-100 positivity confirms a neuroectodermal origin. The lack of synaptophysin positivity makes a diagnosis of neurocytoma unlikely. Other glial neoplasms are made less likely by the classic oligodendroglial morphology seen in this tumor, including round, regular nuclei surrounded by perinuclear halos and interspersed fine, branching capillaries; there was likewise a lack of astrocytic morphology or classic features of ependymoma, such as perivascular pseudorosettes or ependymal canals. Pilocytic astrocytomas may contain areas with oligodendroglioma-like morphology; however, the current tumor lacks the typical piloid processes and biphasic architecture of a pilocytic astrocytoma, and no Rosenthal fibers or eosinophilic granular bodies were identified. DNETs can contain areas resembling oligodendroglial morphology, with small, round cells with perinuclear halos, but these lesions also have patterned nodules and a neuronal component not seen in the present tumor. Another rare entity that should be considered is leptomeningeal oligodenrogliomatosis. The histologic features are that of a classic oligodendroglioma and can have low or high grade features; however, the radiologic appearance consists predominately of focal or diffuse leptomeningeal enhancement as opposed to a mass forming lesion within the cortex [7, 8].

Codeletion of 1p and 19q is a frequent finding in oligodendroglial neoplasms in the adult population but is much less common in pediatric oligodendrogliomas, especially in children less than 10 years old [1, 5, 9, 10]. Recently, Rodriguez et al. examined a group of 50 pediatric patients with oligodendrogliomas; they found that, with rare exception, patients with 1p/19q codeleted tumors were older than 16 at the time of diagnosis. Therefore, the lack of the 1p/19q codeletion in the present case is not unexpected and does not conflict with the diagnosis of congenital oligodendroglioma. Likewise, although adult oligodendrogliomas frequently harbor detectable isocitrate dehydrogenase (IDH1) mutations, these mutations are almost always absent in pediatric gliomas [5, 11, 12]. In the series presented by Rodriguez et al., mutant IDH1 (R132H) immunohistochemistry was only positive in a subset of their 1p/19q codeleted pediatric oligodendrogliomas. Based on these previous reports, the expectation is that congenital gliomas (including congenital oligodendroglioma) would similarly be negative for the mutant specific IDH1 immunohistochemical stain, as was exhibited in the current case.

Structural neuroimaging plays an important diagnostic and prognostic role in children presenting with acute onset epilepsy. Choosing the correct modality early in the disease process provides crucial information regarding etiology and allows identification of lesions requiring surgical intervention. Although MRI is the imaging modality of choice in these cases because of superior anatomic resolution [13–15], computed tomography (CT) is advantageous in the resolution of blood and calcification [13]. Additionally, CT scans are cheaper, more widely available and are less likely to require sedation in young children. Coupled with EEG, as was done in the reported case, CT scan is described as an acceptable screening tool but is not definitive for the resolution of mass lesions [13, 16]. The current case underscores the importance of choosing the most appropriate imaging modality as the described lesion may have been identified earlier in the disease course with MRI.

In conclusion, this case of a low grade oligodendroglioma presenting within the first few months of life represents a rare example of congenital oligodendroglioma and underscores the importance of maintaining these tumors in the differential diagnosis of infants presenting with new-onset seizures. Similar to previously reported pediatric oligodendrogliomas, this congenital tumor was negative for 1p/19q codeletion as well as for R132H mutant specific IDH1 by immunohistochemistry. Though as a group pediatric oligodendroglioma patients have demonstrated good overall survival, particularly in those patients with nonanaplastic tumor histology [5], our experience with congenital oligodendrogliomas remains limited, warranting close clinical and radiologic follow-up for these unique patients.
